# Co-feeding glucose with either gluconate or galacturonate during clostridial fermentations provides metabolic fine-tuning capabilities

**DOI:** 10.1038/s41598-020-76761-4

**Published:** 2021-01-08

**Authors:** Theresah N. K. Zu, Sanchao Liu, Elliot S. Gerlach, Wais Mojadedi, Christian J. Sund

**Affiliations:** 1 Combat Capabilities Development Command Army Research Laboratory, SEDD, Adelphi, MD 20783 USA; 2 Oak Ridge Associated Universities, Belcamp, MD 21017 USA

**Keywords:** Metabolic engineering, Regulatory networks

## Abstract

*Clostridium* *acetobutylicum* ATCC 824 effectively utilizes a wide range of substrates to produce commodity chemicals. When grown on substrates of different oxidation states, the organism exhibits different recycling needs of reduced intracellular electron carrying co-factors. Ratios of substrates with different oxidation states were used to modulate the need to balance electron carriers and demonstrate fine-tuned control of metabolic output. Three different oxidized substrates were first fed singularly, then in different ratios to three different strains of *Clostridium* sp. Growth was most robust when fed glucose in exclusive fermentations. However, the use of the other two more oxidized substrates was strain-dependent in exclusive feeds. In glucose-galacturonate mixed fermentation, the main products (acetate and butyrate) were dependant on the ratios of the substrates. Exclusive fermentation on galacturonate was nearly homoacetic. Co-utilization of galacturonate and glucose was observed from the onset of fermentation in growth conditions using both substrates combined, with the proportion of galacturonate present dictating the amount of acetate produced. For all three strains, increasing galacturonate content (%) in a mixture of galacturonate and glucose from 0 to 50, and 100, resulted in a corresponding increase in the amount of acetate produced. For example, *C.* *acetobutylicum* increased from ~ 10 mM to ~ 17 mM, and then ~ 23 mM. No co-utilization was observed when galacturonate was replaced with gluconate in the two substrate co-feed.

## Introduction

Biology is inherently capable of producing certain high-value products, where synthetic chemistry struggles^1,2^. Synthesis of chemicals using microorganisms requires complex, intensive, and time consuming metabolic engineering. However, chemical production pipelines are executable as discrete components that are quickly inserted or deleted^3,4^. This bioengineering step provides the necessary pathway flux for the desired chemical production. However, it is challenging to know the correct amount of a particular carbon substrate and redox co-factors of the right oxidation state for enabling enzymes of a specific desired pathway in the context of background cellular metabolism. Current available technologies or processes are not amenable to complex inputs^5^. Traditionally, single substrates, most notably sugars, such as glucose, have been explored as substrates, leading to several limitations on the metabolism of cells^6^. In contrast, providing microbes with alternate substrates has the potential to ease such constraints and mitigate issues by adding an extra degree of freedom to the cellular systems and can allow for optimization of fermentation products^6^.

Advances in synthetic biology are unlocking the potential to produce a vast array of chemicals with microorganisms^7–9^. Chemicals production via synthetic pathways imposes a metabolic burden on cells since resources, in the form of carbon and energy, are re-directed towards the desired product^6,10,11^. Maximizing product yields without sacrificing the microbial cells that serve as product factories is essential for sustained and economical chemical production^12,13^. Synthetic and natural pathways require exact proportions of precursors such as carbon, ATP, and electron carriers to produce the desired chemical^6,14^. Generally, product synthesis in cells occurs at varying efficiency levels that involves the coordination of different biological building blocks. In particular, exclusively fed, substrates produce specific carbon-to-energy ratios that are different from carbon-to-energy requirements of desired products^15^. Surplus precursors are metabolically wasteful and deprive the cell of precious resources that could be re-directed toward growth and maintenance^6^. This excess input can lead to a production of undesired products^16,17^. Current strategies at re-direction of these excess inputs have focused on the generation of biosynthetic pathways^18–20^ and the reconfiguration of bioreactors^21^. However, less consideration has gone into the optimization of precursor production in cells. A method to provide exact precursor proportions for synthetic pathways would eliminate metabolic waste and, in turn, increase chemical yields.

Anaerobic clostridia have a long history of acetone, butanol, and ethanol production from carbohydrate-rich substrates in ABE fermentation. Anaerobic fermentation has promise as an alternative solution for energy recycling of food waste, which generates approximately 30–60% of the total solid waste globally^22^. In military environments, challenges with generated food waste impose an additional logistical burden on Army operations^23,24^, with current guidelines including burial or active burning of any generated food waste^25^. These clostridia can utilize a wide range of carbohydrates found in lignocellulosic biomass, which is readily available as agricultural, agro-industrial, and food waste^26^. Plant biomass found in agricultural lignocellulosic biomass contains pectin, a carbohydrate polymer made up of glucose, l-arabinose, and d-galacturonate^27^. d-Galacturonate and d-gluconate (present in fruit) are oxidized carbohydrate derivatives, which have been the focused substrates in previous *C.* *acetobutylicum* studies^28^. To advance food waste fermentation processes, an understanding of the metabolism of these oxidized substrates is essential. Our lab observed homoacetic fermentation on galacturonate in previous research, suggesting a potential route for biologically derived acetic acid production^28^. Advances in genetic engineering techniques^29,30^ in clostridia, during the last decade, have unlocked the potential of inserting synthetic pathways for chemical production into these organisms. The solventogenic clostridia are excellent synthetic biology chassis candidates because of their wide substrate range, high level of carbon flux through acetyl-CoA, and demonstrated ability to produce chemicals at scale^31^.

The high level of flux through acetyl-CoA is particularly important because it can serve as the precursor to the synthesis of many classes of compounds such as acetate, ethanol, lactate, butyrate, butanol, and acetone^32–34^. Co-feeding of substrates to solventogenic clostridium is a possible mechanism to control proportions of ATP, NADH, and acetyl-CoA^35^. A barrier to co-feeding is carbon catabolite repression (CCR), which occurs widely in nature and prevents co-utilization if preferred substrates are present^33^. CCR occurs in the solventogenic clostridium were glucose, and in some cases, arabinose represses the utilization of less preferred carbohydrates^36–38^. Many clostridia can consume substrates that differ from carbohydrates in the oxidation state. These include aldonic acids, uronic acids, sugar alcohols, and glycerol. This capability can be exploited for fine-tuned metabolic control. However, there is a lack of information about the co-utilization of these substrates and regulatory processes, which would prevent co-utilization.

In this study, we examine the ability to fine-tune the metabolism of *Clostridium* sp*.* by co-feeding the organism a mix of glucose together with either of two more oxidized substrates. By varying the proportion of galacturonate in the mixture, it was possible to fine-tune control of reduced electron carrier production, thereby enabling optimization of redox inputs to particular chemical production pathways without genetic alterations. This approach bypasses the limitations of conventional techniques or solutions that generally require pathway modification via metabolic engineering^12^, which tend to be laborious processes without guarantee of the desired output or result in other negative side-effects. The ability to use native pathways to exert metabolic control through the choice of controlled substrate, explained here, decreases the risks and costs associated with direct metabolic engineering.

## Results

### Control of metabolic state of fermentative organisms

**Figure 1:**
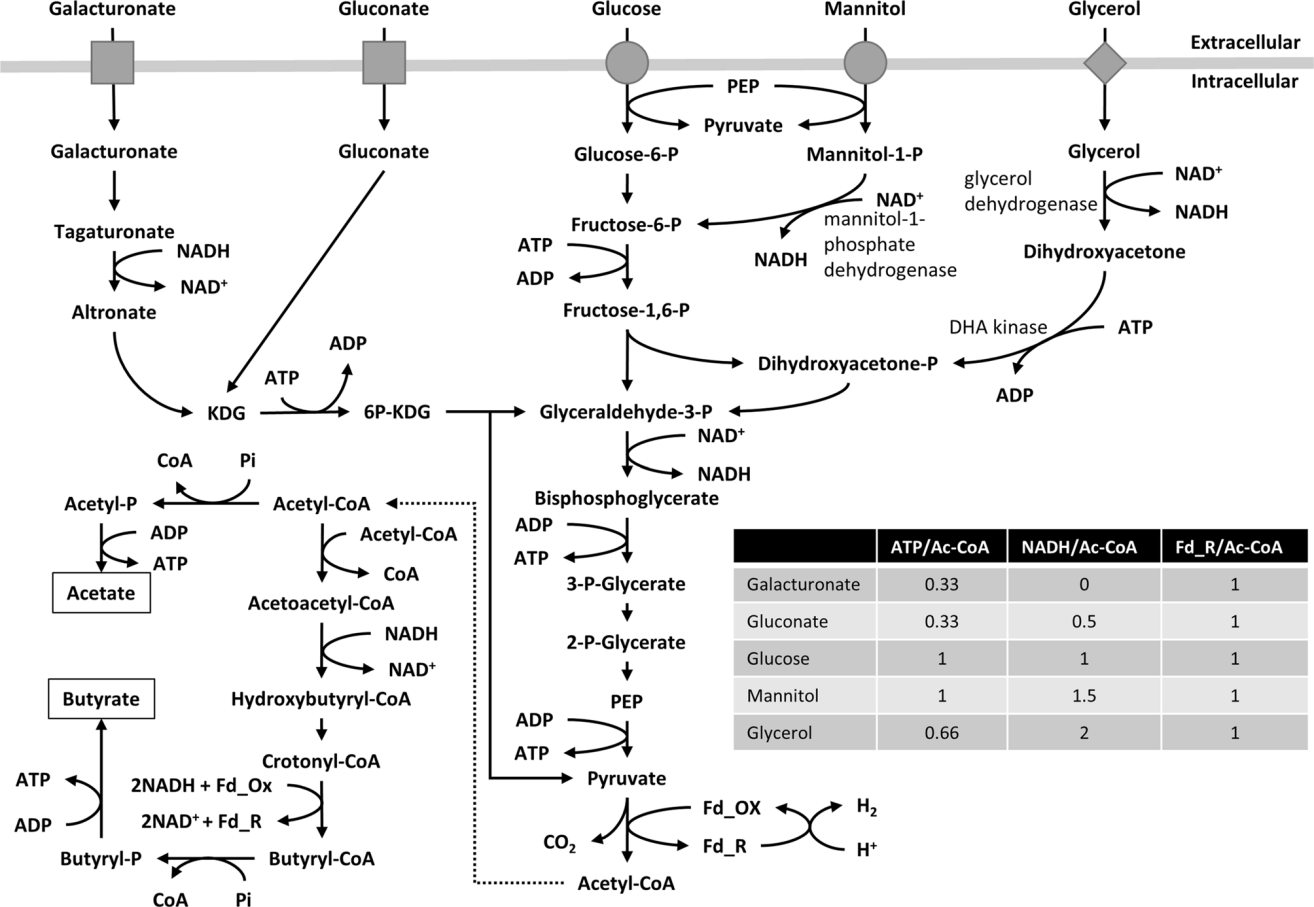
Metabolic network of *Clostridium* *acetobutylicum*. Updated pathway from original published work (Servinsky et al.^28^).

Fermentative organisms such as *Clostridium* sp. adjust their metabolic state to make the most efficient use of substrates with different oxidation states. They do this by altering the recycling of reduced electron carriers such as NADH and NADPH. In general, the higher the oxidation state of a substrate, the less NAD(P)H it generates in its catabolism^35^. During homoacetic fermentation on galacturonate^28^, the batch fermentation was thought to be incomplete regardless of product inhibition or low substrate utilization. Also, galacturonate-grown cells exhibited reduced growth rates when the fermentation culture was agitated, suggesting loss in H_2_ reductant, required for redox balancing. A detailed pathway of these carriers when *C.* *acetobutylicum* is fed three substrates (galacturonate, gluconate, glucose) of different oxidation states is presented in Fig. 1, below. The figure is an updated pathway from an earlier publication which includes pathways for mannitol and gylcerol utilization^38,39^. Figure 1 shows that from uptake to acetyl-CoA a combination of 0.3ATP/0NADH is produced during galacturonate utilization, 0.3ATP/0.5NADH for gluconate, 1ATP/1NADH for glucose, 1ATP/1.5NADH for mannitol, and 0.6ATP/2NADH for glycerol. These ratios thus present discreet points of operation during individual substrate feeding.

### Growth on singular and mixed substrates

One strain, *C.* *acetobutylicum*, was initially tested in experiments for utilization of substrates during the acidogenic phase of the fermentation. *C.* *acetobutylicum* cells were cultured following the 13 substrate combinations (minus combinations of 75%/25%) in Table 1 below. From the OD_600_ (Supplementary Figure S1a) and metabolites data (supplementary Figure S2a–c), individual substrate feeding suggests glucose was the most readily used of the three substrates with cells utilizing (producing) ~ 25 mM glucose (~ 14 mM butyrate, ~ 10 mM acetate), ~ 19 mM gluconate (~ 17 mM acetate and ~ 6 mM butyrate), and ~ 15 mM galacturonate (~ 23 mM acetate as the sole product) within the first 8 h of fermentation. In order of decreasing oxidation state, glucose was the most reduced substrate, followed by gluconate and then galacturonate, with the most oxidized substrate being nearly homoacetic. Gluconate though used more readily than galacturonate, resulted in a substantial decrease in cell growth rate indicated by the OD_600_ data with *C.* *acetobutylicum* being the most tolerant strain. On the other hand, growth on galacturonate was robust for *C. saccharoperbutylacetonicum* and *C. beijerinckii* and less robust for *C.* *acetobutylicum*.

**Table 1:** Combinations of three substrates with different oxidation states for *Clostridium* sp. fermentations. Also shown are the net NADH per acetyl CoA for production of the two main fermentation products - acetate and butyrate. Values for growth on individual substrate feeding are shown in bold, whereas predicted outcomes for co-feeds are shown in italic. In general growth on these substrates resulted in product mixtures required for redox balancing. The negative values indicate that undesirable by-products are generated during this process. So in general, targeted butyrate production will always have associated acetate as a by-product for electron balancing.

Experimental conditions					
	%C in co-feed			Net NADH per acetyl-CoA from substrate to product	
Available substrates	Glucose	Gluconate	Galacturonate	Acetate	Butyrate
#1	100	0	0	1	−**0.50**
#2	75	0	25	*0.75*	−*0.75*
#3	50	0	50	*0.50*	−*1*
#4	25	0	75	*0.25*	−*1.25*
#5	0	0	100	**0**	−**1.50**
#6	0	25	75	*0.13*	−*1.138*
#7	0	50	50	*0.25*	−*1.25*
#8	0	75	25	*0.38*	−*1.13*
#9	0	100	0	**0.50**	−**1**
#10	25	75	0	*0.63*	−*0.88*
#11	50	50	0	*0.75*	−*0.75*
#12	75	25	0	*0.88*	−*0.63*
#13	33.33	33.33	33.33	**0.50**	−*0.99*

When *C.* *acetobutylicum* was co-fed equal amounts of two substrates (Table 1—substrates #3, #7, and #11), growth was substrate dependant (Supplementary Figure S1b, c). Metabolite data (Supplementary Figure S2d) suggest co-feeding equal portions of glucose with gluconate was the less preferred of the two-substrate mixtures, yielding approximately equal proportions of acetate and butyrate at ~ 7.5 mM. However, in co-feeds of equal amounts of glucose with galacturonate (Supplementary Figure S2), approximately twice the amount of acetate (~ 17 mM) was produced compared to butyrate (~ 7 mM). Co-feeding equal amounts of galacturonate with gluconate (Supplementary Figure S2.) produced ~ 23 mM acetate as the main product, comparative to yield from galacturonate only (Supplementary Figure S2c) even though a more considerable amount of the mixed substrate was consumed (~ 25 mM vs. ~ 15 mM from before). When equal proportions of all three substrates were co-fed to *C.* *acetobutylicum* (substrate #13, Supplementary Figure S2g), suppressed growth was observed (data not included), and produced yields of ~ 13 mM acetate and ~ 6 mM butyrate.

### Substrate co-utilization and product regulation in *C. acetobutylicum*

**Figure 2:**
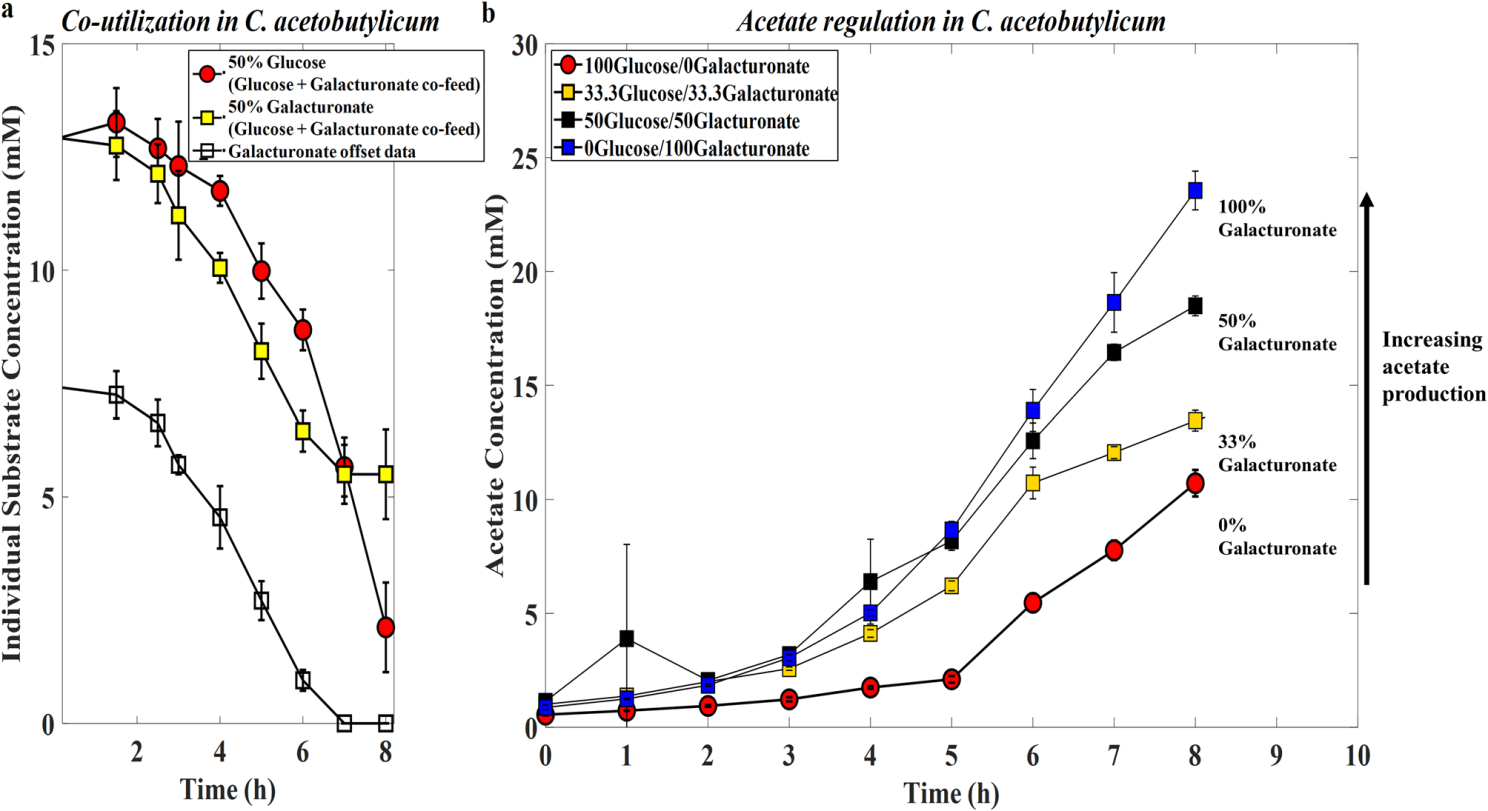
(**a**) Individual substrate consumption when *C.* *acetobutylicum* is co-fed a mixture of glucose and galacturonate. (**b**) Acetate regulation in *C.* *acetobutylicum* based on glucose-galacturonate co-feeding and co-utilization. Error bars are standard deviations of three biological repeat experiments.

Careful examination of the results from the two-substrate growth curves (Supplementary Figure S1b, c), in comparison with individual substrate growth (Supplementary Figure S1a), suggested possible co-utilization of glucose and galacturonate. New experiments were conducted along-side repeat analyses to confirm this hypothesis. Samples were also collected for HPAEC-PAD analyses to determine the individual consumption profile of the different substrates in each mixture. The results presented in Fig. 2a below suggest that during the first 7 h of fermentation, *C.* *acetobutylicum* cells simultaneously co-utilized both glucose and galacturonate until the latter's exhaustion from the mixture. *C.* *acetobutylicum* used all of the galacturonate to completion first when mixed with a more preferred substrate, such as glucose. Combining this knowledge with the fact that galacturonate fermentations are nearly homoacetic, the metabolite data was re-analyzed for possible acetate regulation. Increasing the galacturonate to glucose percent (%) in the feed medium from 0, 50, and then 100 led to increased acetate production. For comparative reasons, co-utilization and acetate production in *C.* *acetobutylicum* are presented side-by-side in Fig. 2. As a positive control, on the same plot, we also plotted the acetate content in the three substrate mixture (#13, 33.3% galacturonate), where only a third of galacturonate was added. The results from Fig. 2b confirmed an increase in the amount of acetate produced (~ 10 mM, ~ 13 mM, ~ 17 mM, and ~ 23 mM) with increasing galacturonate content.

### Fine-tuning metabolite output through substrate co-feeding

The experiments were repeated for two additional species of clostridia*,* *C.* *saccharoperbutylacetonicum* and *C.* *beijerinckii,* to determine whether the observed acetate regulation from glucose-galacturonate co-feeding was species-dependent. We also increased our testing ratios by addition of mid-points (25:75 and 75:25) between exclusive substrate additions (0 and 100), and equal mixtures (50:50) (Table 1—substrates # 2, #4, #8, #10 and #12). Herein also, we extended the experiment time to capture phase transitions from acidogenesis into solventogenesis. OD_600_ measurements for the two species and conditions are presented in Supplementary Figure S1 alongside replicate growth curves for *C.* *acetobutylicum* in the same plot. For both strains, again, glucose had the highest consumption rate (Supplementary Figures S2,S3). In contrast, however, to *C.* *acetobutylicum* fermentation discussed above, galacturonate was consumed more readily than gluconate in exclusive feeds. In exclusive galacturonate experiments, again, fermentations were nearly homoacetic. Also, the two strains appeared intolerant of gluconate as a substrate, producing insignificant amounts of metabolic products.

**Figure 3:**
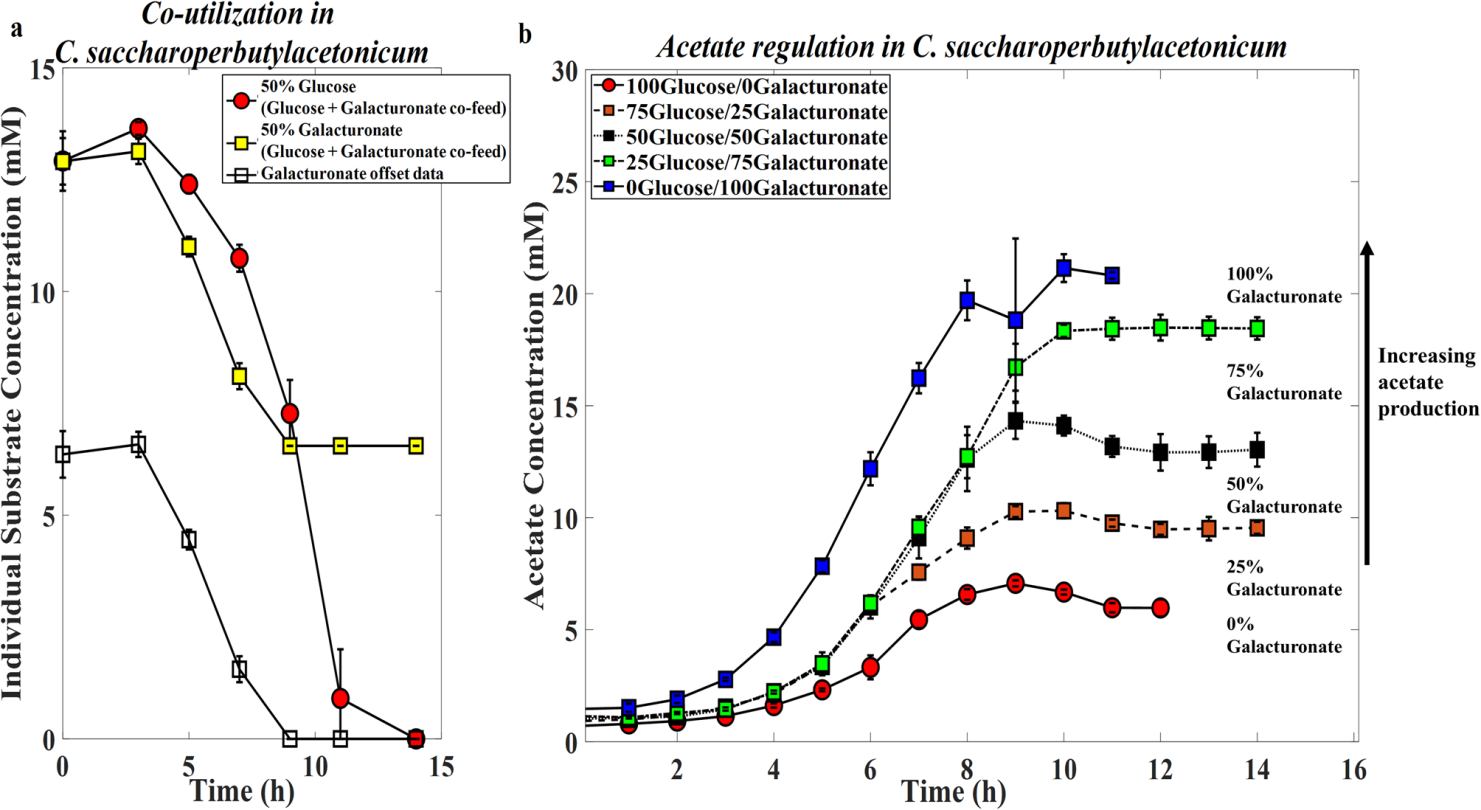
(**a**) Individual substrate consumption when *C.* *saccharoperbutylacetonicum* is co-fed a mixture of glucose and galacturonate. (**b**) Acetate regulation in *C.* *saccharoperbutylacetonicum* based on glucose-galacturonate co-feeding and co-utilization. Error bars are standard deviations of three biological repeat experiments.

The metabolite data from exclusive glucose fermentation (~ 30 mM consumed, Supplementary Figure S3a), show that *C.* *saccharoperbutylacetonicum* produced near equal proportions of acetate (~ 6 mM) and butyrate (~ 5 mM) during acidogenesis, switching to high butanol production (~ 17.5 mM) in the second phase of the fermentation (solventogenic). During exclusive gluconate fermentations (~ 0.5 mM consumed, Supplementary Figure S3b), *C.* *saccharoperbutylacetonicum* barely consumed the available substrate, producing minimal fermentation products (~ 3.5 mM butyrate, and ~ 2.5 mM acetate). However, in exclusive galacturonate fermentations (~ 17.5 mM consumed, Supplementary Figure S3c), *C.* *saccharoperbutylacetonicum* fermentation was mostly homoacetic yielding ~ 21 mM acetate. When *C.* *saccharoperbutylacetonicum* was co-fed equal proportions of two substrate mixtures (all substrates minus #1, #5, and #9), growth was again substrate dependant (Supplementary Figure S1e,f). In co-fed fermentations with glucose-gluconate (Supplementary Figure S1e), growth was similar to exclusively fed glucose fermentation (Supplementary Figure S1d). The comparative metabolite data for the two-substrate mixtures (Supplementary Figure S3) for *C.* *saccharoperbutylacetonicum* are as follows: in general, for substrate mixtures with glucose-gluconate (Supplementary Figure S3d–f) no co-utilization was observed (data not shown), and acetate regulation, if any, was at a minimum from ~ 5 mM to ~ 7.5 mM during the experiment as the gluconate percent content was increased from 25 to 50, and then 75. In co-feeds with glucose-galacturonate (Supplementary Figure S3g–i), co-utilization of glucose and galacturonate was observed within the first 9 h of fermentation (Fig. 3a). Acetate yields increased (Fig. 3b) from ~ 7 mM to ~ 10 mM, ~ 14 mM, ~ 19 mM, through to ~ 21 mM as the galacturonate percent content in the feed was increased from 0 to 25, 50, 75, and 100 respectively. In all co-fed two substrate mixtures (Supplementary Figure S3d–i), the more oxidized substrates likely failed to produce the conditions needed for solventogenesis due to the lower availability of NADH. Substrate combinations with overall lower oxidation numbers were more favorable for butanol production. For example, glucose-gluconate mixtures were more favorable compared to glucose-galacturonate mixtures.

**Figure 4:**
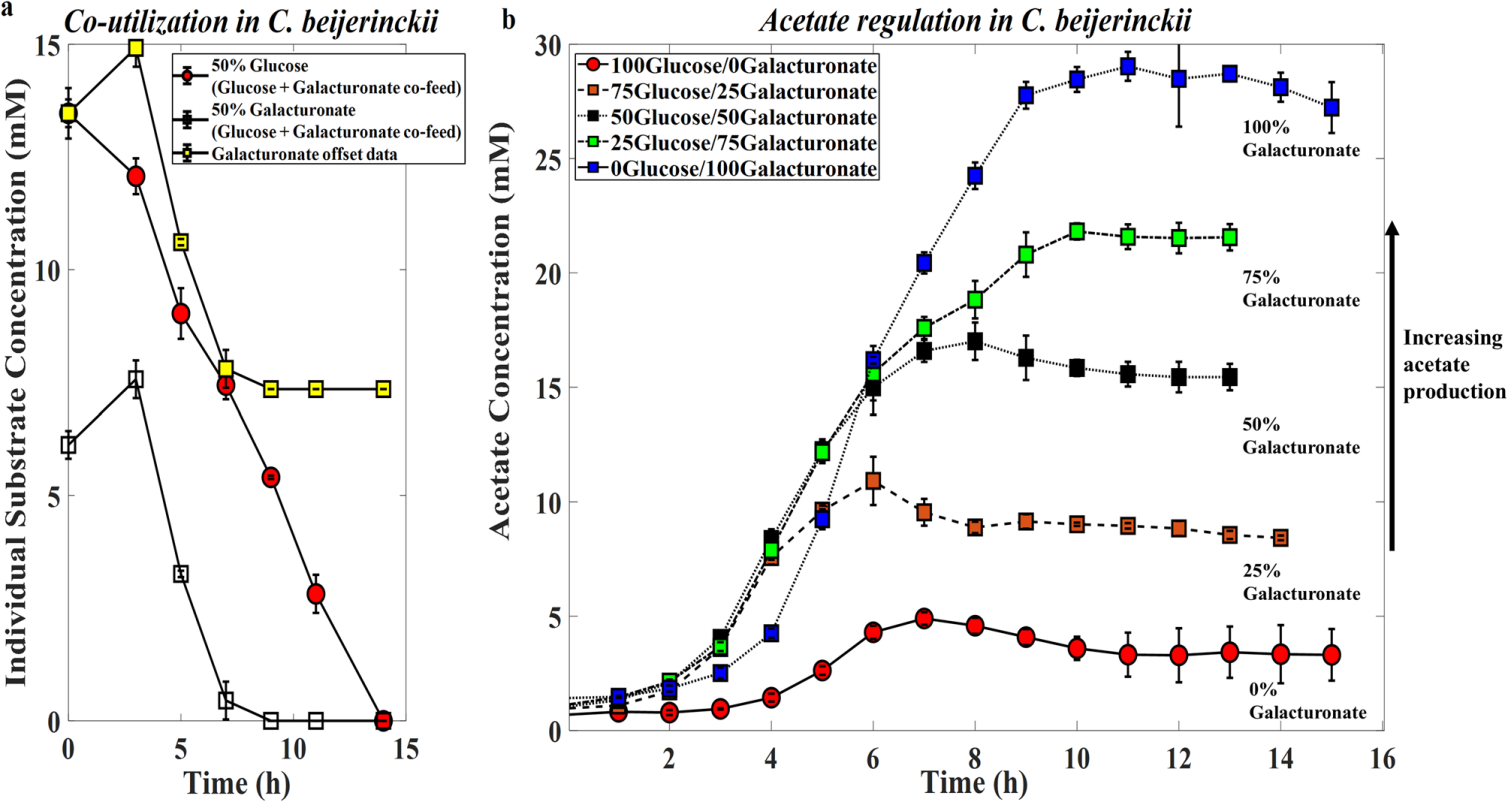
(**a**) Individual substrate consumption when *C.* *beijerinckii* is co-fed a mixture of glucose and galacturonate. (**b**) Acetate regulation in *C.* *beijerinckii* based on glucose-galacturonate co-feeding and co-utilization. Error bars are standard deviations of three biological repeat experiments.

In *C.* *beijerinckii*, the metabolite data for exclusive glucose fermentation (~ 20 mM consumed, Supplementary Figure S4a), show that *C.* *beijerinckii* produced mostly butyrate (~ 17.5 mM) during the 14 h of fermentation. During exclusive gluconate fermentation (~ 0.5 mM consumed in the first 12 h, Supplementary Figure S4b), *C.* *beijerinckii* consumed very minimal substrates and produced insignificant amounts of fermentation products (recorded below ~ 3 mM on average). Exclusive galacturonate fermentation (~ 16 mM consumed, Supplementary Figure S4c), for this same strain, was mostly homoacetic (~ 29 mM acetate produced). When *C.* *beijerinckii* was co-fed two substrate mixtures (all substrates minus #1, #5, and #9), growth was generally comparable to growth during exclusive glucose fermentation (Supplementary Figure S1h, i). The comparative metabolite data for the two-substrate mixtures (Supplementary Figure S4) for *C.* *beijerinckii* are as follows: in general, substrate mixtures with glucose-gluconate (Supplementary Figure S4d–f), acetate regulation was not observed, and butyrate was the main product in all three instances. In substrate mixtures with glucose-galacturonate (Supplementary Figure S4g–i) co-utilization of glucose and galacturonate was observed within the first 7 h of fermentation (Fig. 4a) and acetate yields increased (Fig. 4b) from ~ 4 mM to ~ 9 mM, ~ 15 mM, ~ 22 mM, and finally to ~ 28 mM as the galacturonate percent content in the fed was increased from 0 to 25, 50, 75, and then100, respectively. No butanol production was observed for experiments with *C.* *beijerinckii* under all experimental conditions.

## Discussion

This research builds on findings reported by our lab^28^ for agitated *C.* *acetobutylicum* fermentation on glucose, gluconate, and galacturonate. The agitation of fermentation cultures has been shown to reduce total dissolved gas levels in a system^28^, which is advantageous towards growth for glucose and gluconate runs but not for runs on galacturonate. In the work presented here, under agitated batch conditions, the three substrates were co-fed in turns and together for three different *Clostridium* sp*.*—*acetobutylicum*, *saccharoperbutylacetonicum*, and *beijerinckii*.

For exclusive substrate feeds, glucose was the most readily used subsrate for all three *Clostridium* sp. However, fermentation on gluconate and galacturonate was species-dependent. Both *C.* *saccharoperbutylacetonicum* and *C.* *beijerinckii* consumed galacturonate but not gluconate, *C.* *acetobutylicum* consumed galacturonate and gluconate in these experiments and the earlier report for the same organism^28^. The species-dependent consumption of these substrates was further confirmed in the corresponding OD_600_ plots. The metabolite data for exclusively fed galacturonate fermentations supports the earlier results of homoacetic production for all three investigated strains^28^. Like before, for fermentations with glucose or gluconate, the production of butyrate (as the primary product) was species-dependent. Mainly, *C.* *ace*tobutylicum produced butyrate under both substrate conditions. However, the other two clostridia strains only produced butyrate with glucose consumption and insignificant metabolite products for the less desirable substrate—gluconate—in this instance.

When galacturonate was co-fed with glucose its presence correlated to increased acetate production for all three species. The main finding in this work is the product regulation of acetate. In part, these results are supportive of research by Vasconcelos et al.^35^, where metabolic output is affected by glucose-glycerol ratios in two-substrate co-feeds. In this very instance, glucose is the more oxidized substrate in comparison to glycerol.

**Figure 5:**
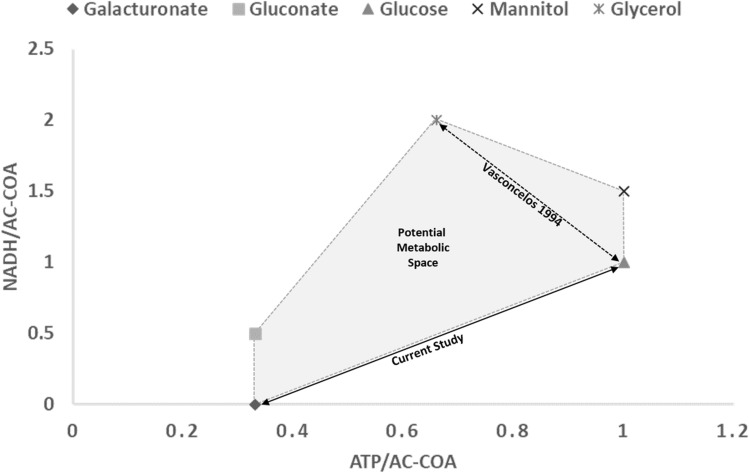
Proposed continuous exploration for chemical outputs from fermentations. Plot shows ratio of ATP/NADH that is produced per each acetyl-CoA formed.

Substrate co-utilization presents several advantages and opens up a vast region of exploration for control of chemical production in biological systems. Instead of access to discrete points shown in Fig. 1, substrate co-utilization via manipulation of glucose and galacturonate in different proportions can enable advanced chemical production through a biological route. In addition to the three substrates (galacturonate, gluconate, and glucose) explored in this work, more substrates with different oxidation states (e.g., mannitol and glycerol) can be explored for possible co-utilization. The current findings show a proof of concept for continuous control of precusors for chemical production denoted by the plot in Fig. 5 above as opposed to discrete points which result from single substrates. For example, using *C.* *acetobutylicum* as a case study, co-feeding equal ratio of glucose and galacturonate in the two-substrate mixtures had the same acetyl-CoA (~ 0.5) as exclusive feed (~ 0.5) on gluconate. However, it produced twice the amount of ATP (~ 0.7 vs. ~ 0.35). Since the redox recycling requirements remained unchanged, the acetate: butyrate ratios (2:1) are comparable in both instances (Supplementary Figure S2b,e). However, the carbon flux, and consequently, product yields differ under these two scenarios. The observed higher carbon flux results in more biomass due to ATP yields and subsequently lower products for the co-fed equal ratio of glucose and galacturonate. In exclusive gluconate feeds, however, higher product yields result in less biomass, likely because more subsrate is needed to make equivalent ATP. The directional carbon flux demonstrates another dimension of metabolic control beyond the redox changes explained via the acetate and butyrate ratio. By extending this analogy, a similar amount of ATP (~ 0.7) was produced for equal ratio co-feeds of glucose with either gluconate or galacturonate. However, NADH/acetyl-coA requirements were different (~ 0.5 vs. ~ 0.75). The change in redox recycling requirements is reflected in the acetate:butyrate ratios produced (1:1 vs. 2:1), as shown in Supplementary Figure S2,e. For tri-equal co-feed for galacturonate, gluconate, and glucose (33:33:33), NADH/acetyl-coA requirements (~ 0.5), and associated ATP production (~ 0.7) was similar to that for equal ratio co-feed of glucose and galacturonate. Again we see similar or un-changed acetate:butyrate ratio (2:1—Supplementary Figure S2g).

## Conclusion

In order to bypass limitations on the degree of metabolic freedom, researchers should embrace the more complex bio-products synthesis employing multiple substrates in a fermentation. Traditionally, single substrates have been employed but have been shown to be metabolically wasteful and, in most instances, produce undesirable products, thus depriving the cell of precious resources that could be re-directed toward growth and maintenance. In this research, we show that desirable metabolic products can be regulated and optimized by co-feeding a mixture of substrates with different oxidation states to *Clostridium* sp*.,* where substrates co-utilization is observed.

We present an exploratory region for five substrates with varying degrees of oxidations: galacturonate, gluconate, glucose, mannitol, and glycerol and show that acetate: butyrate ratios can be regulated through co-feeding (if co-utilization is present in the chosen organism). Likewise, we demonstrate another dimension of metabolic control beyond the redox, where carbon flux can be tuned toward higher product yields based on NADH/acetyl-CoA and ATP prevalence.

Future discoveries of co-utilization of other substrate mixes (e.g., for the five substrates listed above) can further open up this exploratory region. Additionally, different anaerobic fermentating bacteria can also be explored as candidates for this space. In silico modeling and simulations can be used together with a design of experiments to minimize the cost and time needed to narrow down potential candidates for subsequent testing and practical validation in the laboratory.

## Materials and methods

### Experimental design

Three (3) substrates: glucose, galacturonate, and gluconate were chosen as feedstock for fermentation experiments based on their oxidation states and reported metabolites. Clostridia cells were grown on the three individual substrates and several combinations of the substrates, as shown in Table 1 above.

### Bacteria strain maintenance and anaerobic culture conditions

*Clostridium* *acetobutylicum* ATCC 824, *Clostridium* *saccharoperbutylacetonicum* ATCC 27021 and *Clostridium* *beijerinckii* ATCC 25752 were purchased from the American Type Culture Collection (ATCC) and used in all experiments. Strains were maintained as spore stocks in a suspension of potato glucose medium containing per liter DI water: potato (grated), 150.0 g; Glucose, 10.0 g; (NH_4_)_2_SO_4_, 0.5 g; and CaCO_3_, 3.0 g combined, then boiled 1 h mixing every 10 min, filtered through cheesecloth, and autoclaved 120 °C 10 min exposure^11^. Spore stocks were stored at room temperature in an anaerobic chamber (Coy Lab Products, MI) containing an atmosphere of approximately 90% N_2_, 5% CO_2_, and 5% H_2_.

Clostridial Growth Medium (CGM) was composed of the following components per liter of DI water: KH_2_PO_4_, 0.75 g; K_2_HPO_4_, 0.75 g; MgSO_4_·H_2_O, 0.4 g; MnSO_4_, 0.01 g; FeSO_4_·7H_2_O, 0.01 g; NaCl, 1.0 g; l-asparagine, 2.0 g; yeast extract, 5.0 g; (NH_4_)_2_SO_4_, 2.0 g; substrate (glucose, gluconate, or galacturonate), 5.0 g^11^. Chemicals used in medium preparation were purchased from Sigma Aldrich (St. Louis, MO, USA) and Becton Dickinson (Franklin Lakes, NJ, USA) and of HPLC grade (99%), or extract suitable for use in culture media preparations.

*Clostridium* sp. was initially propagated on CGM under anaerobic conditions described previously^28,40^. 500 µL of potato-glucose suspension (PGM) spore stock was heat-shocked at 80 °C for 10 min, then re-suspended in 150 mL CGM enriched with 0.5% of the respective carbohydrate (i.e., glucose, galacturonate, gluconate, or mixtures two or three of these substrates).

### HPLC sample acquisition and analysis—individual sugars and metabolites

Cell growth experiments were conducted in triplicate for each of the three species investigated. 1 mL samples were extracted from experimental cultures hourly for OD_600_ measurements (Ultrospec 10/Amersham Biosciences). An additional 1 mL sample extracted, sterile filtered through a 0.2 µm syringe filter (Corning), then stored at + 4 °C for metabolite analysis via HPLC. HPLC analysis for the metabolic output was completed using an Agilent 1200 equipped with a refractive index detector and an Aminex HPX-87H cation exchange column (300 × 7.8 mm i.d. × 9 µm; Bio-Rad)^41^. 100 µL of each sample was injected into the system for isocratic elution, with a mobile phase of 3.25 mM H_2_SO_4_ at 0.6 mL/min and 35 °C.

### HPLC sample acquisition and analysis—sugar mixtures

In order to confirm the co-utilization of glucose with galacturonate, new separation methods for detection were developed and used. The sugar mixtures were separated by high-performance anion-exchange chromatography with pulsed amperometric detection (HPAEC-PAD). The chromatography was carried out on a Dionex DX-600 system consisting of an AS50 autosampler, LC30 column compartment, GP50 gradient pump, and ED50 electrochemical detector (Dionex/Thermo Fisher Scientific). The column is CarboPac PA1 (4 mm × 250 mm). The injection volume is 10 µL. The gradient is shown below in Table 2.

**Table 2:** HPAEC-PAD separation parameters.

Time (min)	%A	%B	%water
00.00	80	0	20
20.00	80	0	20
50.00	0	100	0
60.00	0	100	0
61.00	80	0	20
75.00	80	0	20

A: 15 mM NaOH; B: 150 mM NaOAc in 15 mM NaOH. Flow rate: 1 mL/min. Column temperature: room temperature.

## Supplementary information

Supplementary Information.
