# Supplementary material for: Co-feeding glucose with either gluconate or galacturonate during clostridial fermentations provides metabolic fine-tuning capabilities
Source: Sci Rep. 2021 Jan 8;11:29. doi: 10.1038/s41598-020-76761-4 (PMC7794554; doi:10.1038/s41598-020-76761-4)
Supplement: Supplementary file 1 — Supplementary Information. [file 41598_2020_76761_MOESM1_ESM.pdf]

Co-feeding glucose with either gluconate or galacturonate during clostridial fermentations provides metabolic fine-tuning capabilities.

Theresah N.K. Zu[1, *], Sanchao Liu[1, +], Elliot S. Gerlach[1, +], Wais Mojadedi[2], and Christian J. Sund[1]

[1]Combat Capabilities Development Command Army Research Laboratory, SEDD, Adelphi, MD 20783, USA
[2]Oak Ridge Associated Universities, Belcamp, MD 21017, USA
[*]Correspondence to [Theresah.Zu.civ@mail.mil]
[+]These authors contributed equally to this work

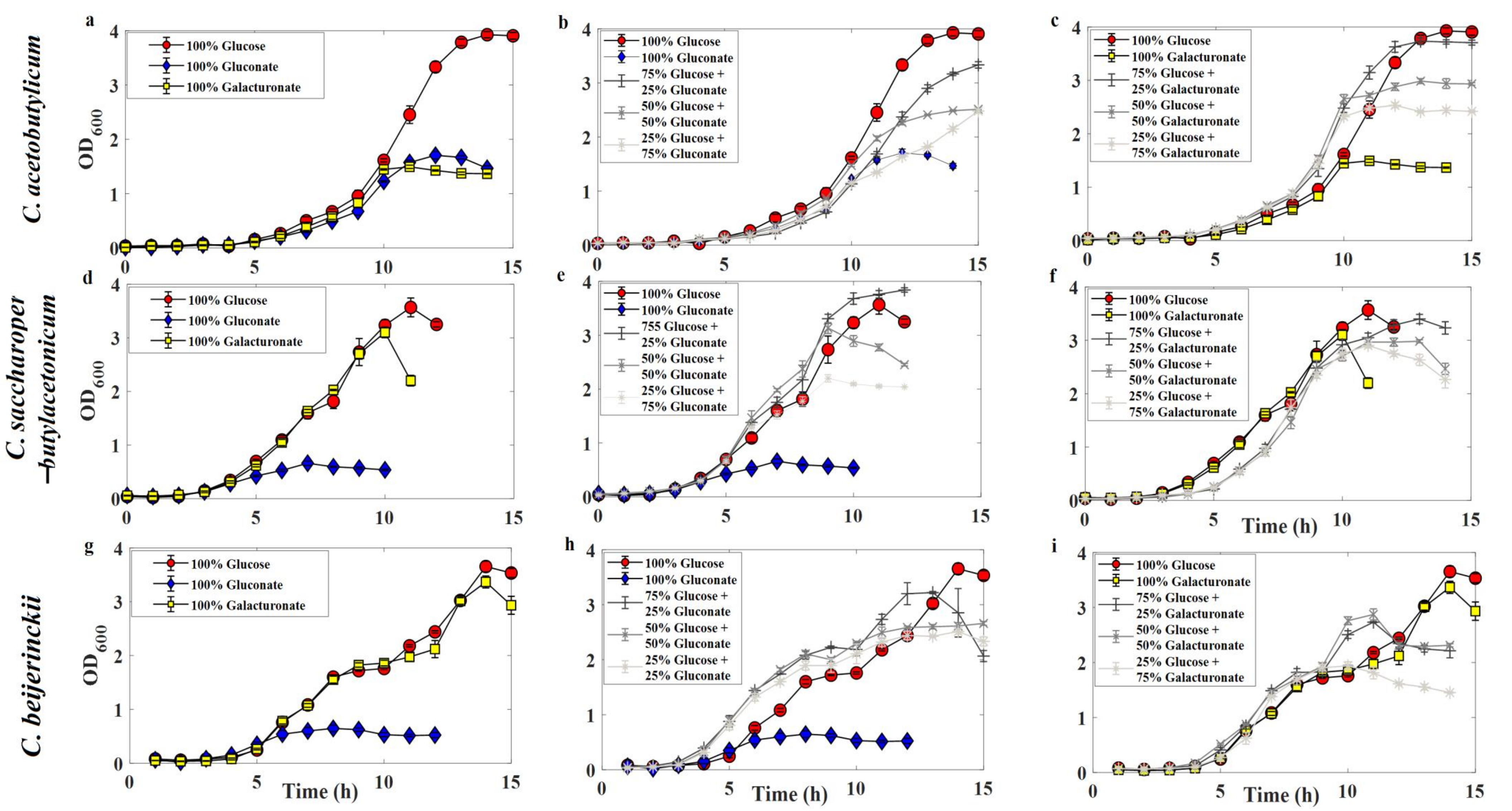
C. acetobutylicum
C. saccharoper
–butylacetonicum
C. beijerinckii
a
b
c
d
e
f
g
h
i
OD600
Time (h)
100% Glucose
100% Gluconate
100% Galacturonate
75% Glucose +
25% Gluconate
50% Glucose +
50% Gluconate
25% Glucose +
75% Gluconate
75% Glucose +
25% Galacturonate
50% Glucose +
50% Galacturonate
25% Glucose +
75% Galacturonate
755 Glucose +
25% Gluconate
25% Glucose +
25% Gluconate
0
1
2
3
4
5
10
15

Supplementary Figure 1. Comparative optical density (OD_{600}) measurements for fermentations of three *Clostridia sp*. – *acetobutylicum (a – c), saccharoperbutylacetonicum (d – f), beijerinckii (g – i)* - fed exclusively on glucose, gluconate or galacturonate (a, d, g); mixtures of glucose and gluconate (b, e, h); and glucose and galacturonate (c, f, i) per table 1. Error bars are standard deviations of three biological repeat experiments

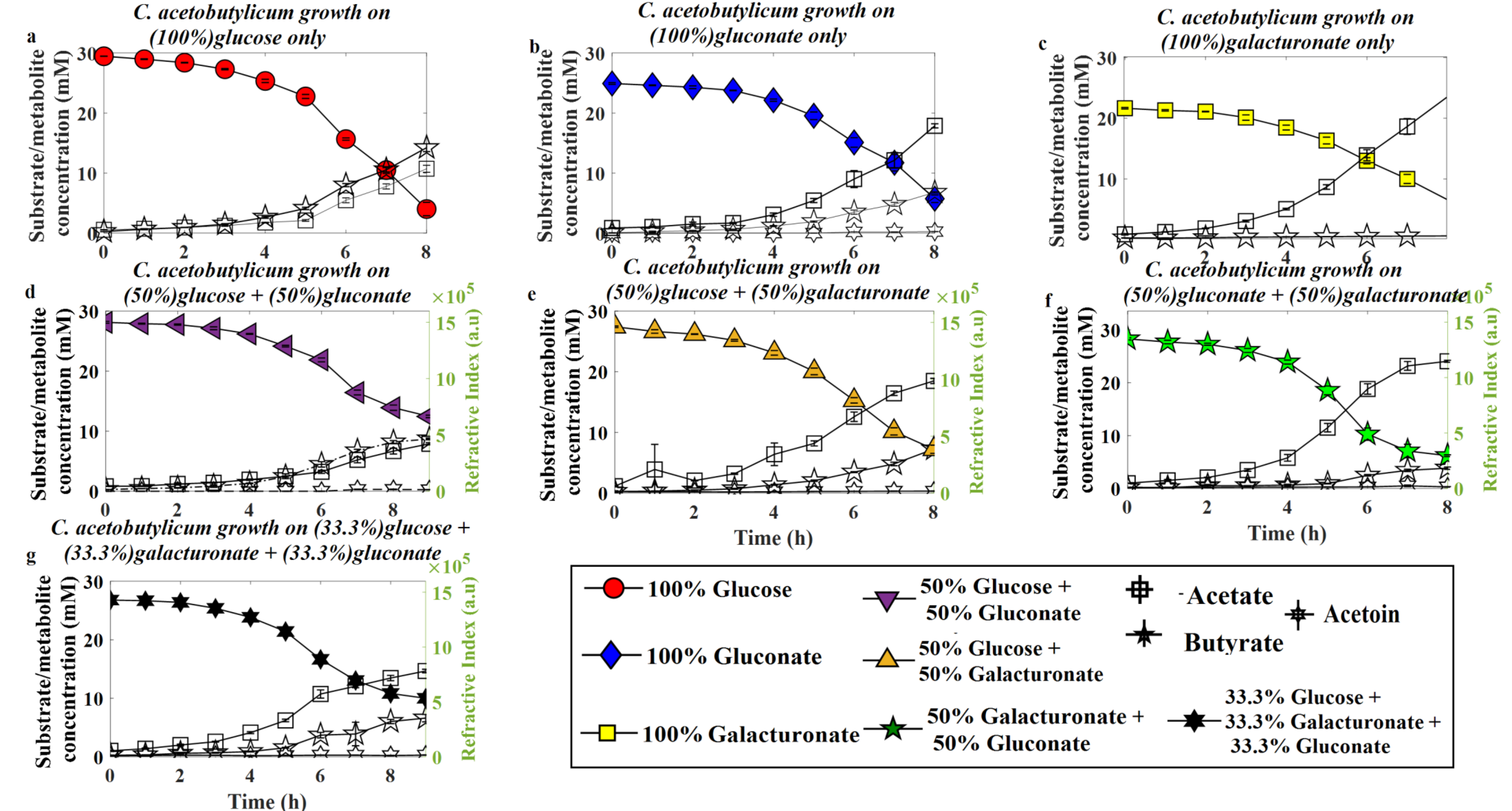
a
C. acetobutylicum growth on (100%)glucose only
b
C. acetobutylicum growth on (100%)gluconate only
c
C. acetobutylicum growth on (100%)galacturonate only
d
C. acetobutylicum growth on (50%)glucose + (50%)gluconate
e
C. acetobutylicum growth on (50%)glucose + (50%)galacturonate
f
C. acetobutylicum growth on (50%)gluconate + (50%)galacturonate
g
C. acetobutylicum growth on (33.3%)glucose + (33.3%)galacturonate + (33.3%)gluconate
Substrate/metabolite concentration (mM)
Refractive Index (a.u)
×10^5
Time (h)
100% Glucose
100% Gluconate
100% Galacturonate
50% Glucose + 50% Gluconate
50% Glucose + 50% Galacturonate
50% Galacturonate + 50% Gluconate
Acetate
Butyrate
Acetoin
33.3% Glucose + 33.3% Galacturonate + 33.3% Gluconate

Supplementary Figure 2. Metabolites profile when *Clostridia acetobutylicum* was fed solely on glucose (a), gluconate (b), galacturonate (c), or an equal mixture of glucose and gluconate (d), an equal mixture of glucose and galacturonate (e) an equal mixture of gluconate and galacturonate (f) or equal mixtures of glucose, galacturonate and gluconate (g) per table 1. Error bars are standard deviations of three biological repeat experiments

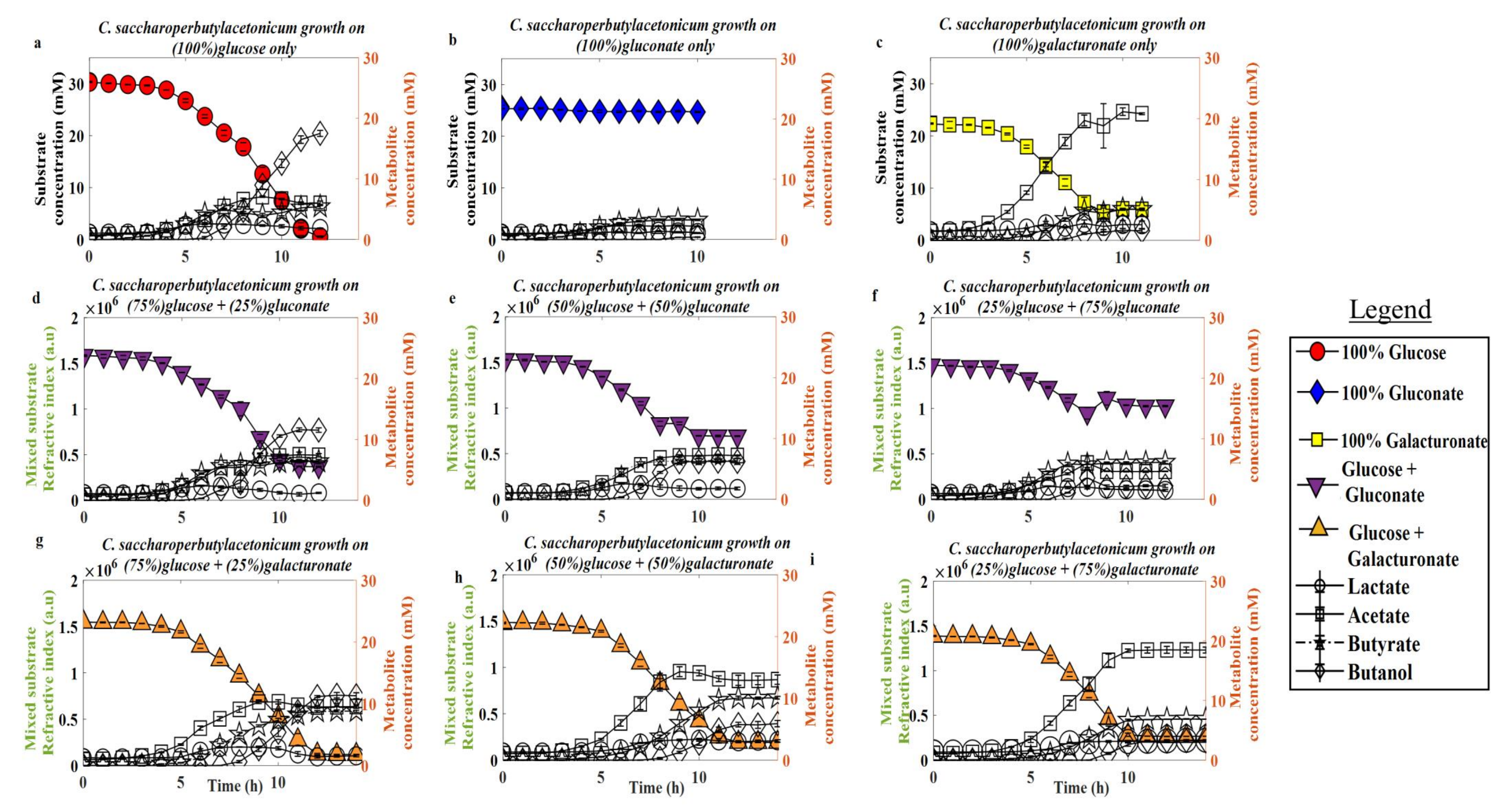

a
C. saccharoperbutylacetonicum growth on (100%)glucose only
b
C. saccharoperbutylacetonicum growth on (100%)gluconate only
c
C. saccharoperbutylacetonicum growth on (100%)galacturonate only
Substrate concentration (mM)
Metabolite concentration (mM)
d
C. saccharoperbutylacetonicum growth on (75%)glucose + (25%)gluconate
e
C. saccharoperbutylacetonicum growth on (50%)glucose + (50%)gluconate
f
C. saccharoperbutylacetonicum growth on (25%)glucose + (75%)gluconate
g
C. saccharoperbutylacetonicum growth on (75%)glucose + (25%)galacturonate
h
C. saccharoperbutylacetonicum growth on (50%)glucose + (50%)galacturonate
i
C. saccharoperbutylacetonicum growth on (25%)glucose + (75%)galacturonate
×10^6
Mixed substrate Refractive index (a.u)
Time (h)
Legend
100% Glucose
100% Gluconate
100% Galacturonate
Glucose + Gluconate
Glucose + Galacturonate
Lactate
Acetate
Butyrate
Butanol

Supplementary Figure 3. Metabolites profile when *Clostridia saccharoperbutylacetonicum* was fed solely on glucose (a), gluconate (b), galacturonate (c), or mixtures of glucose and gluconate (d, e, f), or mixtures of glucose and galacturonate (g, h, i) per table 1. Error bars are standard deviations of three biological repeat experiments

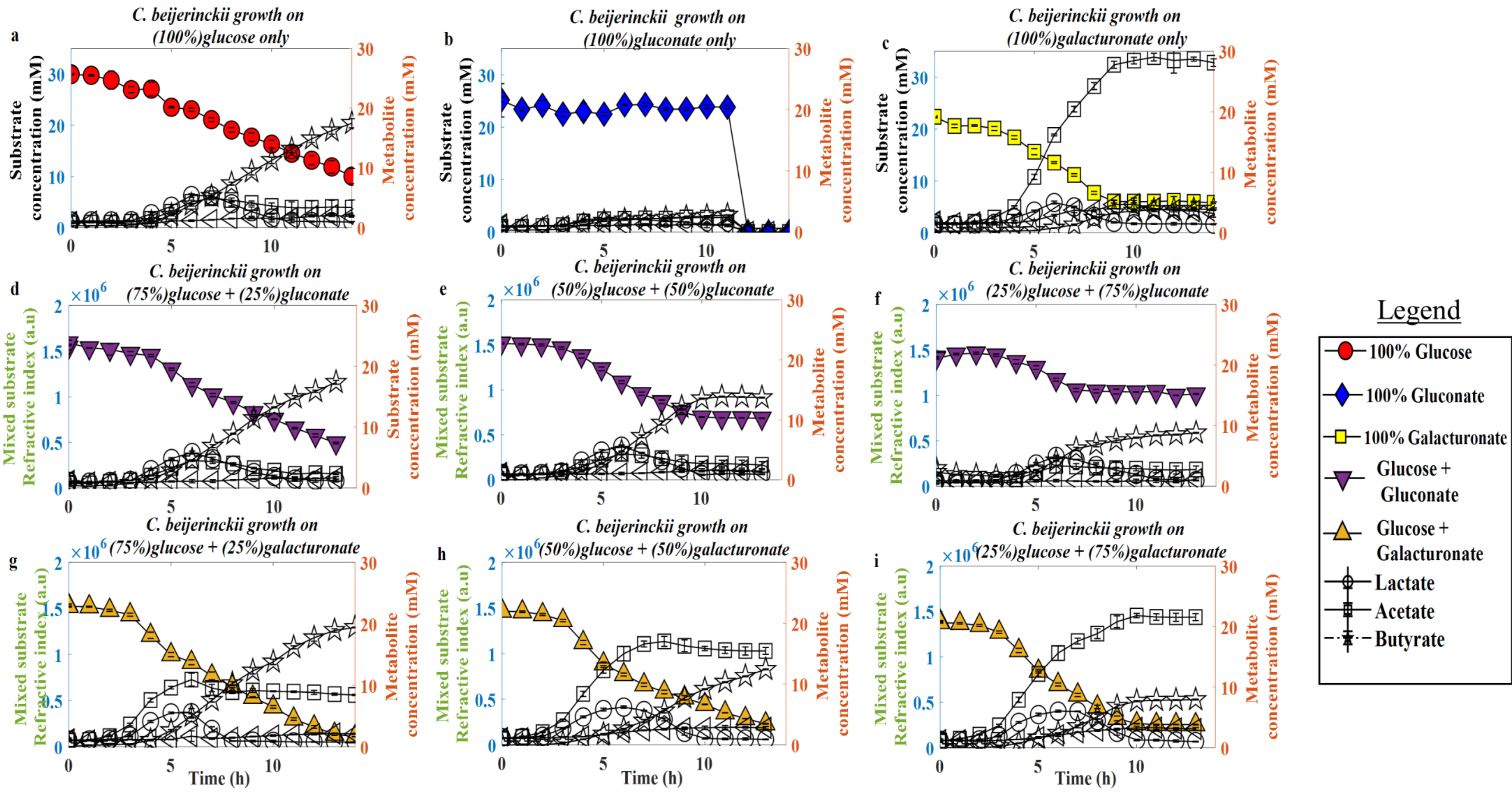

a
C. beijerinckii growth on (100%)glucose only
Substrate concentration (mM)
Metabolite concentration (mM)
b
C. beijerinckii growth on (100%)gluconate only
c
C. beijerinckii growth on (100%)galacturonate only
d
C. beijerinckii growth on (75%)glucose + (25%)gluconate
Mixed substrate Refractive index (a.u)
Substrate concentration (mM)
e
C. beijerinckii growth on (50%)glucose + (50%)gluconate
f
C. beijerinckii growth on (25%)glucose + (75%)gluconate
g
C. beijerinckii growth on (75%)glucose + (25%)galacturonate
h
C. beijerinckii growth on (50%)glucose + (50%)galacturonate
i
C. beijerinckii growth on (25%)glucose + (75%)galacturonate
Time (h)
×10^6
Legend
100% Glucose
100% Gluconate
100% Galacturonate
Glucose + Gluconate
Glucose + Galacturonate
Lactate
Acetate
Butyrate

Supplementary Figure 4. Metabolites profile when *Clostridia beijerinckii* was fed solely on glucose (a), gluconate (b), galacturonate (c), or mixtures of glucose and gluconate (d, e, f), or mixtures of glucose and galacturonate (g, h, i) per table 1. Error bars are standard deviations of three biological repeat experiments

Supplementary Figure 1. Comparative optical density (OD_{600}) measurements for fermentations of three *Clostridia sp*. – *acetobutylicum (a – c), saccharoperbutylacetonicum (d – f), beijerinckii (g – i)* - fed exclusively on glucose, gluconate or galacturonate (a, d, g); mixtures of glucose and gluconate (b, e, h); and glucose and galacturonate (c, f, i) per table 1. Error bars are standard deviations of three biological repeat experiments

Supplementary Figure 2. Metabolites profile when *Clostridia acetobutylicum* was fed solely on glucose (a), gluconate (b), galacturonate (c), or an equal mixture of glucose and gluconate (d), an equal mixture of glucose and galacturonate (e) an equal mixture of gluconate and galacturonate (f) or equal mixtures of glucose, galacturonate and gluconate (g) per table 1. Error bars are standard deviations of three biological repeat experiments

Supplementary Figure 3. Metabolites profile when *Clostridia saccharoperbutylacetonicum* was fed solely on glucose (a), gluconate (b), galacturonate (c), or mixtures of glucose and gluconate (d, e, f), or mixtures of glucose and galacturonate (g, h, i) per table 1. Error bars are standard deviations of three biological repeat experiments

Supplementary Figure 4. Metabolites profile when *Clostridia beijerinckii* was fed solely on glucose (a), gluconate (b), galacturonate (c), or mixtures of glucose and gluconate (d, e, f), or mixtures of glucose and galacturonate (g, h, i) per table 1. Error bars are standard deviations of three biological repeat experiments
